# The Welfare of Fighting Dogs: Wounds, Neurobiology of Pain, Legal Aspects and the Potential Role of the Veterinary Profession

**DOI:** 10.3390/ani12172257

**Published:** 2022-08-31

**Authors:** Daniel Mota-Rojas, Chiara Mariti, Míriam Marcet-Rius, Karina Lezama-García, Angelo Gazzano, Ismael Hernández-Ávalos, Patricia Mora-Medina, Adriana Domínguez-Oliva, Alexandra L. Whittaker

**Affiliations:** 1 Neurophysiology, Behavior and Animal Welfare Assessment, DPAA, Universidad Autónoma Metropolitana (UAM), Mexico City 04960, Mexico; 2 Department of Veterinary Sciences, University of Pisa, 56124 Pisa, Italy; 3 Animal Behaviour and Welfare Department, IRSEA (Research Institute in Semiochemistry and Applied Ethology), Quartier Salignan, 84400 Apt, France; 4 Facultad de Estudios Superiores Cuautitlán, Universidad Nacional Autónoma de México (UNAM), Mexico City 54714, Mexico; 5 School of Animal and Veterinary Sciences, Roseworthy Campus, University of Adelaide, Roseworthy, SA 5116, Australia

**Keywords:** dog fight, dog welfare, illegal sports, animal fight, pain, dog aggression

## Abstract

**Simple Summary:**

Dog fights are cruel and harmful events which have a clear impact on animal welfare. For this reason, many countries have banned these events via statute. However, in some regions of the world they are still legal. Moreover, the enforcement of legal bans can be problematic in countries where they are illegal, and they may still occur. This article provides background information on dog fighting and the welfare implications of it. This includes consideration for the pain inflicted, and its mechanisms of perception and recognition. It also analyzes the injuries and emotions experienced by the animals and considers the profile of the breeders and handlers involved in the activity. Since welfare concerns often extend beyond the animals’ fighting lives, a discussion around the possibilities of reintroduction into suitable environments for these animals is also made. Finally, attention is turned to the role that veterinarians can and should play in dealing with these issues of welfare.

**Abstract:**

Throughout history it has been common to practice activities which significantly impact on animal welfare. Animal fighting, including dogfighting, is a prime example where animals often require veterinary care, either to treat wounds and fractures or to manage pain associated with tissue and where death may even result. Amongst the detrimental health effects arising are the sensory alterations that these injuries cause, which not only include acute or chronic pain but can also trigger a greater sensitivity to other harmful (hyperalgesia) or even innocuous stimuli (allodynia). These neurobiological aspects are often ignored and the erroneous assumption made that the breeds engaged in organized fighting have a high pain threshold or, at least, they present reduced or delayed responses to painful stimuli. However, it is now widely recognized that the damage these dogs suffer is not only physical but psychological, emotional, and sensory. Due to the impact fighting has on canine welfare, it is necessary to propose solution strategies, especially educational ones, i.e., educating people and training veterinarians, the latter potentially playing a key role in alerting people to all dog welfare issues. Therefore, the aim of this review is to describe the risk factors associated with dogfighting generally (dog temperament, age, sex, nutrition, testosterone levels, environment, isolation conditions, socialization, education, or training). A neurobiological approach to this topic is taken to discuss the impact on dog pain and emotion. Finally, a general discussion of the format of guidelines and laws that seek to sanction them is presented. The role that veterinarians can play in advancing dog welfare, rehabilitating dogs, and educating the public is also considered.

## 1. Introduction

In various regions of the world, organized fights involving animals, such as bulls, camels, horses, dogs, cocks, crickets, and rams, are popular events [1]. In many countries, they have now been banned. However, despite increasing public awareness and concern for animal welfare, there is still a proportion of the population that flout these laws and continue to organize and attend dog fights [2]. Organized dog fights are activities in which two dogs, usually of the same sex and weight, are provoked to fight as a form of entertainment or means of making money by betting [3]. The rings used for dog fights can measure 12, 16, or 20 feet per side [4]. Fights continue with the dogs injuring each other until one is exhausted or dies [5]. Some authors suggest that dog fights are related to organized crime [6], drug- or arms-trafficking, animal abuse, and illegal gambling [7], among other types of violence [5,8].

In the last decades, animal welfare has emerged as a priority for many people and organizations [9], across all areas of animal use, with particular attention often given to companion animals due to the often special nature of the human–animal bond with this group of animals. Animal fights usually fall into two categories: (1) intra-species fights, including species such as dogs, cocks, beetles, and other insects, and (2) inter-species fights (combinations between species) [10]. Both are practices that inflict intentional and severe abuse to animals with no regard to their welfare. Although “blood sports” and animal fighting are usually used as synonymous, some blood sports do not include fighting (e.g., foxhunting) and are legal activities that end with the death of the animal [11]. Many countries have recently reformed animal protection laws to make the organization and attendance of animal fights illegal. However, in spite of these laws, which are commonly associated with severe penalties, there is evidence that dog fighting continues with significant animal welfare cost. For example, estimates from the United States suggest that despite these legal bans, 16,000 dogs are still raised for organized fights and that 44 of them die every day during fights, even though the penalty for organizing them is 5 years in prison or a fine of USD 250,000 [12].

The aim of this review is to provide an overview of dog fighting to help inform policy development. It will consider the risk factors predisposing animals to engage in dogfights, for example character of the dog, age, and sex amongst others. Welfare costs from fighting will be discussed with a particular focus on the neurobiological aspects of pain. Finally, discussion on policy around the activity, the position of the veterinary profession regarding this issue, the psychological profile of breeders or handlers, and current rehabilitation techniques for dogs that have been used in fights will be presented.

## 2. Material and Methods

**Figure 1:**
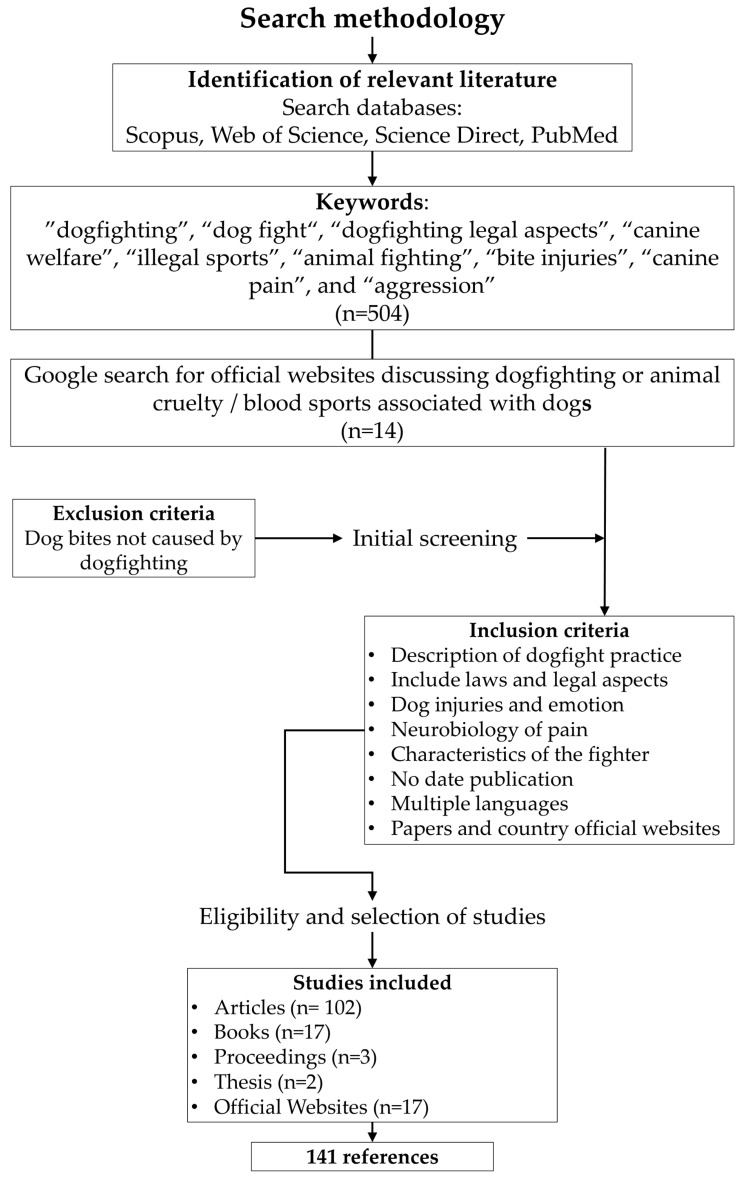
Search methodology.

The review is presented as a structured review, which aimed to identify scientific and grey literature across the scope of this topic. The search was conducted in Scopus, Web of Science, Science Direct, and PubMed databases. The identification of relevant articles was performed using the following keywords: “dogfighting”, “dog fight″, “dogfighting legal aspects”, “canine welfare”, “illegal sports”, “animal fighting”, “bite injuries”, “canine pain”, and “aggression”. Included studies were articles related to the description of dog fight practice and methodology in several countries, laws and legal aspects, emotions in dogs, programs for dog rehabilitation, injuries caused during organized or spontaneous dog fights, and the typology of fighters. There was no settled date of publication. The search was conducted in multiple languages, selecting articles and governmental official websites in English and Spanish. The exclusion criteria included papers where dog injuries were caused by reasons not related to dogfighting and papers regarding animal cruelty associated with other instances. The search methodology and the selection of the 141 references, from 1973 to 2021, for this review are described in Figure 1.

## 3. Cultural-Demographic Characteristics and the Development of Aggressive Tendencies in Dogs

Dog fights are relatively frequent in some Asian countries. They are very popular in Pakistan [12], for example, as are other kind of animal fights. Dog fights are not common in India but are held in some rural localities. In Japan, they are still legal [2,13]. In many countries they are illegal but still occur as illegal activities, for example the US, UK, and Australia.

One of the most popular breeds raised for fights is the Pit Bull Terrier, due to their great strength, apparent tolerance for pain, agility, and evident desire to continue fighting despite the injuries they suffer [8]. That is why the possession of this breed is prohibited in countries, such as Spain, Germany, England, France, Switzerland, Norway, and Denmark [14]. Some evidence suggests that certain breeds present more aggressive behaviors than others. However, as found by Miller et al. [15], not all dogs seized from dogfighting are aggressive with other dogs, this could be because the dog is in its early stages of training and therefore may still preserve non-aggressive social behaviors towards other dogs. Nevertheless, from a scientific and epidemiological point of view it would be risky to argue that any specific breed of dog is more likely to bite people or other animals [16], because so many factors influence the development of an animal’s character: environment, sex, age, education or training, nutrition, isolation, and socialization, among others [17].

Diverse studies show that aggression levels are higher in male dogs than females [18,19,20]. Moore et al. [21], for example, conducted a study that analyzed the prevalence of aggression and biting in dogs of different sexes, reproductive status, and breed. They reported that 87% of the animals that had bitten a person were males, and that 60% of those males had not been castrated. According to Lockwood [22], this could reflect the higher testosterone levels in males, which breeders may increase by administering testosterone propionate to dogs that are bred to participate in organized fights.

Contrary to common thinking, there is evidence that neutered dogs do not reduce their aggressive tendencies under certain circumstances. This argument was analyzed by Neilson et al. [23], who evaluated 57 males that were castrated in an attempt to reduce their aggression before they reached two years of age. They found that only 30% of the animals reduced that behavior, while the other 70% continued to manifest it. Likewise, Farhoody [24] compared the behavior of spayed and non-neutered males, reporting that the aggression levels were greater in the neutered dogs. Observations of females in studies by Guy et al. [25] found the same results. However, it appears that most animals used for dog fighting are unneutered.

In a study by Reid and Collins [26], it was reported that 291 of 292 dogs seized from illegal fights were intact animals ranging from six months to 10 years of age. Interestingly, all the animals were medium-sized dogs with physical dysfunctions (e.g., loss of a limb or an eye) derived from the fights. A similar observation was reported by Montrose et al. [27], who found that out of 151 cases of dog-on-dog aggression, 59.4% of the events were initiated by a medium-sized dog. In this case, the mentioned animals are known to have been genetically selected by humans to show high levels of aggression towards conspecifics. In this sense, Schilder et al. [28] mention the probability that some breeds show greater aggressive behavior towards conspecifics due to genetic factors, although this has not been fully studied. Additionally, having been previously attacked by another dog may be a triggering factor for the aggressive animal to develop aggressive–killing behaviors.

Likewise, according to McGreevy et al. [29], there is a high correlation between small dogs and undesirable behaviors, such as aggression. When Duffy et al. [30] applied a questionnaire to dog-owners (Canine Behavioral Assessment Research Questionnaire C-BARQ), they found that small and medium-size animals showed a greater tendency to commit acts of defense or aggression. This reality is not reflected in studies of the adoption of dogs as pets, as people prefer small dogs over medium-size or large ones. In addition, the belief persists that intact male dog are more aggressive [31] and, as a result, are more often selected for dogfights [12].

## 4. Injuries Caused by Bites

**Figure 2:**
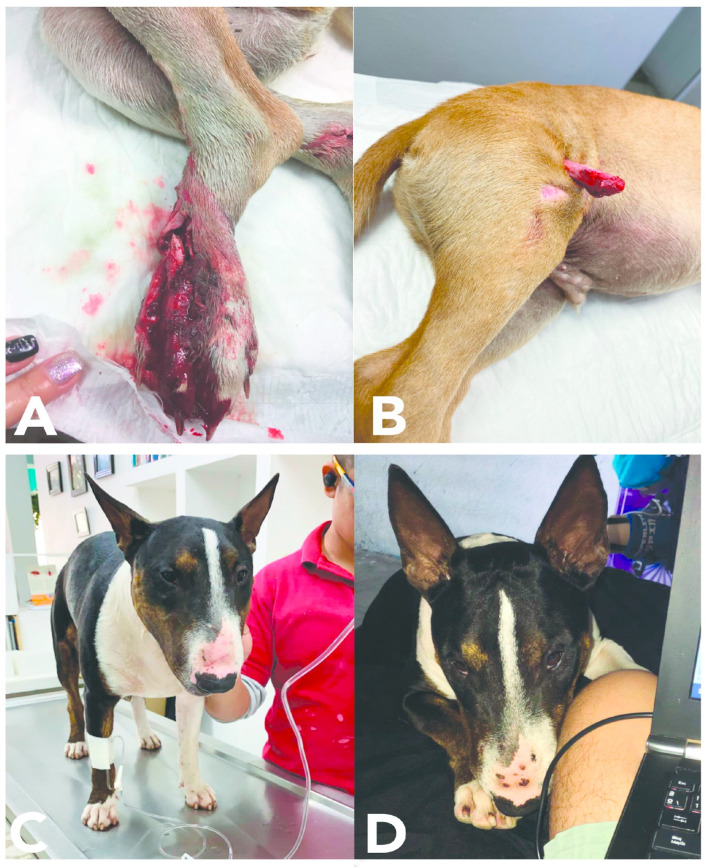
Diverse lesions caused by fights with conspecifics. (**A**) A severe wound in the foot of the left pelvic limb of this patient after a fight with a Rottweiler. The site shows exposed bone (metatarsals, phalanges) and ligaments and the loss of tissue continuity with multiple exposed fractures. (**B**) Image of an exposed fracture of the distal portion of the femur caused by a fight with a stray dog. (**C**,**D**) Images of an English Bull Terrier brought for examination with diverse lesions caused by a spontaneous fight with a stray dog. There are evident scratches and penetrating bites in the muzzle, nasal plane, and left thoracic limb.

**Figure 3:**
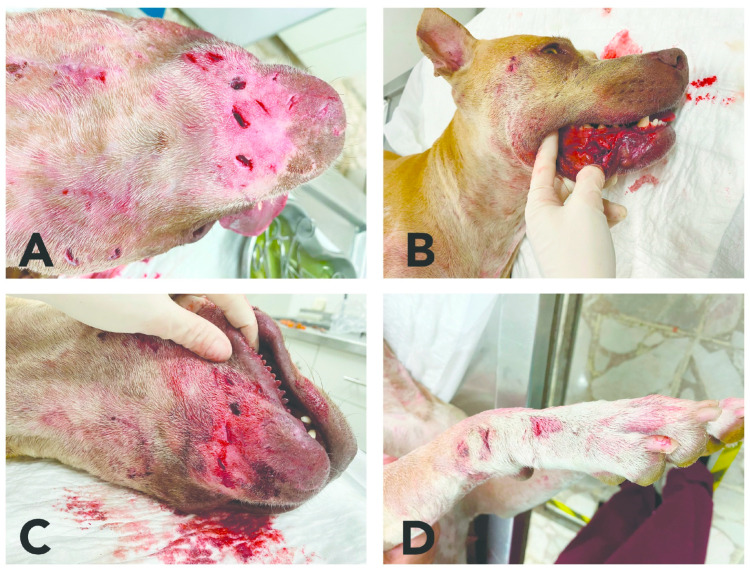
Lesions caused during a fight between two Pit Bulls (one male, one female). The female suffered the deepest wounds, shown in image (**A**,**C**). Obvious orifices in the skin of the mandibular region due to penetrating injuries inflicted by the other dog’s teeth (**B**). Lesions in the oral cavity, specifically the lower gum and lip (**D**). Deep lesions in the right thoracic limb that resulted from bites by the other dog. Most of these lesions required thorough washing and suturing to join the edges of the bites and prescriptions for antibiotics and analgesics.

The injuries that dogs suffer during fights include deep puncture wounds, fractured bones, broken teeth or dental trauma [32], and wounds to the buccal and gingival mucosa (see Figure 2 and Figure 3) dehydration, and infections. There are even reports of cases in which after a dog fight, a fistula was observed in one of the injured dogs, secondary to the presence of a foreign body embedded in the radius region, which turned out to be a fragment of a tooth from the other dog that bit it [33]. Those injuries heal with difficulty and damage the general condition of the dog [34], and if not treated opportunely or adequately, these lesions can cause death [35]. Most penetrating wounds are caused by bites and frequently involve the chest wall [36]. In many fight cases there is also penetration of the body wall to cause damage to the internal organs [37].

Other sequelae include laryngeal paralysis secondary to cervical bite wounds [38]. Broken teeth are also reported both as a result of the fight and from the use of wedge-shaped sticks, which handlers use to separate dogs when they become “fanged”—when the lip of a dog gets caught in its own or its opponent’s teeth. This stick use can result in slab fractures of the canine teeth as well as torn lips [39].

**Figure 4:**
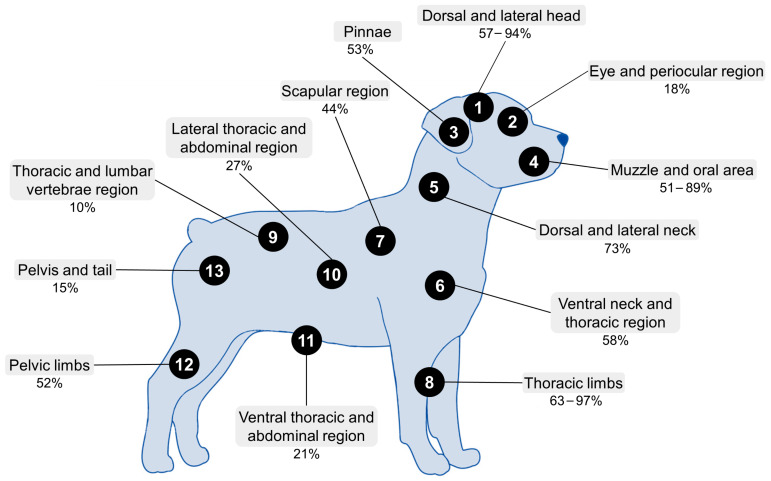
Corporal regions and percentage of the incidences of the principal lesions that occur during dogfights. 1. Dorsal and lateral area of the head with an incidence of 57–94%. 2. Ocular and periorbital region with an incidence of 18%. 3. Pinnae, incidence = 55%. 4. Muzzle and oral mucosa, incidence = 51–89%. 5. Dorsal and lateral aspects of the neck, incidence = 73%. 6. Ventral neck and thoracic region, incidence = 58%. 7. Scapular region, incidence = 44%. 8. Thoracic limbs, incidence = 63–97%. 9. Thoracic and lumbar vertebral region, incidence = 10%. 10. Lateral thoracic and abdominal region, incidence = 27%. 11. Ventral thoracic and abdominal region, incidence = 21%. 12. Pelvic limbs, incidence = 52%. 13. Pelvis and tail, incidence = 15% (Miller et al. [15] and Intarapanich et al. [5]).

Wounds may be characterized into seven types: abrasion, hemorrhage, contusion, laceration, incision, avulsion, and artifact—the latter when a piece of flesh is bitten off the body of the victim [40]. However, a commonly used classification proposed by Griffin [41] is used which categorizes wounds into four classes according to the type and size: the first two as laceration with or without penetration of the dermis and the last two as simple puncture wounds or with an affectation of underlying tissues. Those injuries in the neck and chest are commonly severe [42] and aggression is often directed towards the front of the body. A study by Intarapanich et al. [5] described the principal zones where dogs receive lesions during fights (Figure 4). A study of 252 dogs by Miller et al. [15] found that the lesions and scars observed in animals occurred in the following proportions: 63% in the front legs, 57% in the dorsal and lateral regions of the head, and 51% in the muzzle and oral mucosa.

The main consequence of the wounds is death from blood loss, dehydration, and infection. [43] However, there may also be disease transmission, for example of canine babesiosis [44]. Although most dogs do not fight until death, mortality often occurs after the fight [45].

Because these injuries are often not reported to a veterinarian or detected by the authorities, breeders with no veterinary expertise often provide the only treatment their dogs receive [46].

Whilst it is difficult to differentiate between injuries caused by a spontaneous fight and those that result from organized dog fights, observations suggest that the lesions most commonly inflicted during spontaneous fights occur in the dogs’ shoulders, scruff, and haunches. In contrast, the injuries caused in organized fights tend to affect primarily the head, chest, and forelegs [5]. Another way to discern whether lesions are caused by organized fights is if the animal presents diverse wounds in different stages of healing, as this would indicate it has been exposed to fights on various occasions [8,47]. Scars are characterized by multiple alopecic areas [40]. Additionally, if these types of lesions are recurrent in the same patient it may suggest a fighting history. In dogs presenting for the first time at a clinic, obtaining a history from previous veterinarians may assist in determining involvement in fights.

In addition to the fatal consequences observed in animals involved in dogfighting, damage to tissues and other visceral and bone structures induce a pathophysiological response associated with pain that, in most cases, is not properly treated by a licensed veterinarian, predisposing the animal to further complications.

### 4.1. Neurobiology of Pain in Dogs during Fights

The breeds of dogs trained for organized fights are described as stoic animals that have a high pain threshold or, at least, present reduced responses to painful stimuli [48]. There are cases where dogs have kept on fighting although both of their thoracic limbs were fractured, dragging their chest along the ground to confront their opponent [4]. These behaviors could be due to peculiarities in the opioid receptors or other neurotransmitters. However, as far as we know, there are no published studies with a design that allows us to discuss the relationship between the behavior of fighting dogs and serum levels of beta-endorphin, serotonin, cortisol, or catecholamines as occurs in other species. However, given evidence from other species, including pain biology research in humans, some hypotheses can be derived based on general principles. Due to the existing phylogenetic relationship between species, the work carried out by Fox and Andrews [49] allows a comparison with aggression in wolf cubs. The mentioned study identified differences in the levels of corticosteroids associated with aggression. Those authors evaluated the cardiac frequency of the animals biometrically and analyzed this parameter to detect reactions and emotions. Later, the cubs were confined for a time and stressed by means of handling. Then blood samples were drawn before and after administering ACTH. The cubs that were higher in the social hierarchy showed a greater sympathetic tone and a higher adrenal–pituitary response to stress than the subordinate ones [49].

**Figure 5:**
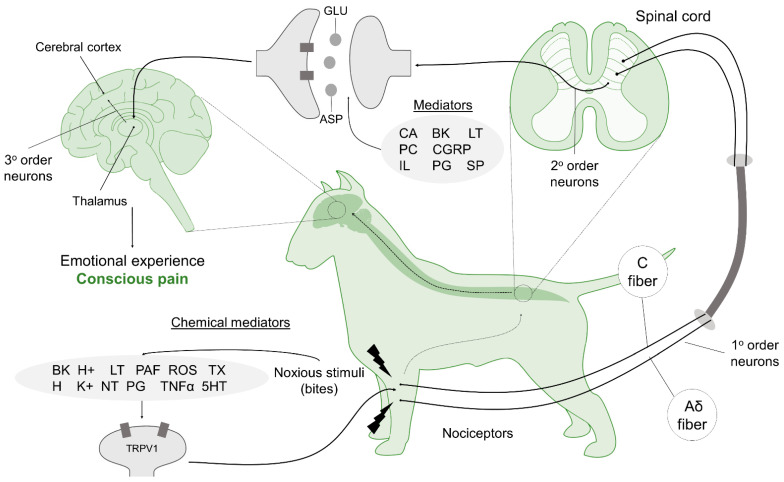
Route of nociceptive transmission from the peripheral nerves to the CNS, beginning with transduction of a nociceptive stimulus during a dogfight. Abbreviations: ASP: aspartate; BK: bradykinin; CA: catecholamines; CGRP: calcitonin-gene-related peptide; GLU: glutamate; H: histamine; H^+^: hydrogen; IL: interleukin; K^+^: potassium; LT: leukotrienes; NT: neurotrophins; PAF: platelet-activating factor; PC: prostacyclin; PG: prostaglandin; ROS: free radicals; SP: substance P; TNFα: tumoral necrosis factor; TRPV1: transient receptor potential vanilloid 1; TX: thromboxane; 5HT: serotonin Johnson et al. [50], Hernández-Avalos et al. [51], Imlan et al. [52], Youn et al. [53], and Mota-Rojas et al. [54]).

Figure 5 shows the route of nociceptive transmission and how acute pain is generated from the skin, muscles, arteries, veins, and connective tissue (where lesions are produced, in this case by bites), all of which contain nociceptors that produce electrical impulses. These signals are send to the central nervous system (CNS), where the organism detects them as pain [50,51,52,53,54] with the participation of the reticular formation to the thalamus, thalamus, and cerebral cortex [55]. From there, the sensation is conducted along the afferent nerves to the CNS [56].

The key components that constitute the perception of pain are described as transduction, transmission, modulation, projection, and perception (see Table 1). Whilst there has been little direct study of these phases in fighting dogs, extrapolation from other species can be made given the similarity in pain biology.

**Table 1:** Phases and characteristics of the nociceptive pathway caused by bite injuries in dogs.

Phases	Description	Main Involved Structures	Chemical Mediators	References
1. Transduction	Transforming harmful stimuli into an electrical impulse (action potential)	∘ Nociceptors in the skin, muscles, bones, and viscera. ∘ Ionic channels (TRP)	5-HT, BK, H, H^+^, K^+^, LT, NT, NY, PAG, PG, ROS, TNF-α, TX	[50,53,54,55,56,57,58,59]
2. Transmission	Electrical impulse travels to the DRG and spinal cord interneurons	∘ Aδ and C free nerves ∘ Spinal cord ∘ Laminae I, II, and V	GLU, SP, CGRP	[57,58,59,60,61]
3. Modulation	Facilitate or inhibit nociceptive transmission	∘ Dorsal horn of the spinal cord ∘ Descending pathways ∘ NMDAr, AMPAr	ASP, BK, CA, CGRP, EOP, IL, LT, PC, PG, SP	[54,61,62,63]
4. Projection	Carry nociceptive inputs from the spinal cord to supraspinal structures	∘ Spinal cord, brainstem, thalamus, hypothalamus, amygdala ∘ Ascending pathways (spinothalamic tract)	GLU, SP	[62,63]
5. Perception	Integration of the conscious recognition of pain	∘ Somatosensory cortex	GLU, SP	[51,55]

AMPAr: AMPA receptor; ASP: aspartate; BK: bradykinin; CA: catecholamines; CGRP: calcitonin-gene-related peptide; DRG: dorsal root ganglion; EOP: endogenous opioid peptides; GLU: glutamate; H: histamine; H^+^: hydrogen; IL: interleukin; K^+^: potassium; LT: leukotrienes; NMDAr: NMDA receptor; NT: neurotrophins; PAF: platelet-activating factor; PC: prostacyclin; PG: prostaglandin; ROS: free radicals; SP: substance P; TNFα: tumoral necrosis factor; TRP: transient receptor potential; TX: thromboxane; 5-HT: serotonin.

### 4.2. Sensitization of the Nervous System

The injuries inflicted in dogfights often force handlers to seek veterinary treatment to treat the dogs’ wounds, set fractures and, above all, control the pain caused by tissue lesions suffered during this activity. Clinical observations confirm that the sensory alterations caused by the typical injuries that occur during fights include continuous pain. By reference to other literature it is proposed that this may become a constant, prolonged stimulus that can increase an animal’s sensitivity to harmful stimuli (hyperalgesia) or even trigger painful responses to innocuous stimuli (allodynia) [64,65].

The inflammation provoked by trauma and tissue lesions means that cytokines, chemokines, growth factors, ATP, H^+^, K^+^, and prostaglandins (PG) are recruited to the site of injury. Peripheral nociceptors and spinal neurons can be sensitized and exhibit greater excitability. In chronic processes, such as the lesions inflicted during dogfights, or when injuries are not treated adequately, exacerbated pain responses are observed in both the injured region (primary hyperalgesia) and surrounding the lesion site (secondary hyperalgesia). In addition, the low threshold of the A fibers causes their recruitment into the ascending modulation of the pain, even though under normal conditions they do not respond to harmful stimuli. During the phenomena of sensitization, however, they participate in the event known as allodynia, when an innocuous stimuli causes pain [62].

**Figure 6:**
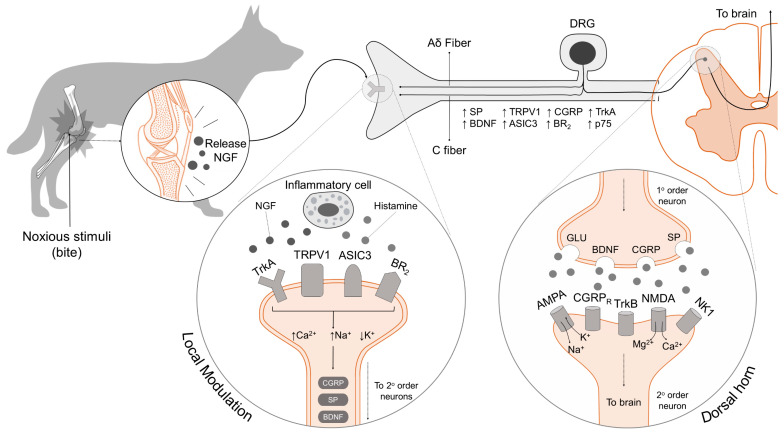
Sensitization of the nervous system. Inflammatory mediators (histamine, NGF) released after tissue injury (bites) bind to peripheral receptors located on first-order neurons. Activation of these receptors (TrkA, TRPV1, ASIC3, BR_2_) and ionic channels, such as Na^+^, Ca^2+^ and K^+^, modulates and enhance the excitability of the primary afferent nerve cells. When the nociceptive signal reaches the dorsal horn of the spinal cord, it causes the release of pronociceptive neurochemicals including GLU, SP, CGRP, and BDNF, from the primary neurons to the second-order neuron receptors, mainly AMPA, NMDA, NK1, CGRPR, and TrkB. These receptors promote the transmission of the excitatory signaling to the brain and cortical structures, where the perception of pain is consciously recognized. Abbreviations: AMPA: α-amino-3-hydroxy-5-methyl-4-isoxazolepropionic; ASIC3: acid-sensing ion channel 3; BDNF: brain-derived neurotrophic factor; BR_2_: bradykinin receptor 2; CGRP: calcitonin-gene-related peptide; DRG: dorsal root ganglion; GLU: glutamate; NGF: nerve growth factor; NK1: neurokinin 1 receptor; NMDA: N-methyl-D-aspartate; p75: neurotrophin receptor; SP: substance P; TrkA: tropomyosin kinase receptor A; TRPV1: transient potential receptor vanilloid 1. DeLeo [66], Verri et al. [67], Watkins et al. [68], and Enomoto et al. [69].

Generally speaking, central sensitization and peripheral sensitization have similar alterations. Glutamate, substance P, and BDNF activate signaling pathways in the dorsal horn through NMDA receptor (Figure 6). The phosphorylation of this receptor increases its response to excitatory neurotransmitters by eliminating voltage-dependent Mg. This process of central sensitization is associated with chronic pain after a lesion to a peripheral nerve or a fracture, as can occur during dogfights. For example, glia produce proinflammatory cytokines, such as IL1B, TNFα, and IL-6, that, together with chemoattractant cytokines, generate the state of increased pain sensation [66,67,68,69].

### 4.3. Adaptive Mechanisms as a Result of the Painful Experience

Findings have shown that wind-up and central sensitization are events present during chronic pain conditions. Wind-up is known as a process of the frequency-dependent and temporal summation of nociceptive input that causes an increased sensation of pain [70]. When injured tissues send pain signals frequently, it causes the overstimulation of nociceptors and surrounding nerve fibers resulting in a potentiation of the pain [71]. In dogs involved in dog fights, they are not only exposed to continuous injuries due to fighting but many of the injuries caused are not treated or are treated inadequately, influencing the presentation of this phenomenon. Since this mechanism facilitates and amplifies the transmission of pain, it also prevents correct modulation of pain [72]. Chronic pain, associated with the wind-up phenomenon, is a clear welfare impost. Additionally, it interrupts the normal functionality of the pain-modulating systems. The hyperactivity of descending facilitatory system or functional impairment of descending inhibitory pathways cease to work and modulate pain in chronic conditions [73]. Some authors have reported that the impairment of these systems makes conventional analgesic therapies insufficient to control this type of pain and more specialized protocols are required [72]. Therefore, when an animal reaches this point after being constantly exposed to lesions in peripheral, bone, or visceral structures, difficult-to-treat chronic pain can occur and is associated with greater pain intensity and emotional consequences in dogs.

Many dogs used for fighting come from establishments that house and raise them exclusively in cages. These animals are exposed to frequent injuries and constant pain that are detrimental to their physical and mental health. McMillan [74] reported that dogs from high-volume commercial breeding establishments have a higher incidence of emotional and behavioral disturbances, such as aggression, fear, and anxiety. These effects may be attributed to early-life adversity, including positive stimulus deprivation, stress, and negative human–animal interaction. In the same way, dogs that are diagnosed with anxiety-related behavioral syndromes have often experienced a lack of daily exercise and other impoverished environments. These events affect their later growth and development [75]. It has been recognized that dog coursing and fighting cause long-term behavioral consequences requiring considerable rehabilitation if those dogs are to go on to live normal lives. This has been something that groups such as the RSPCA and ASPCA have had to address [76]. The latter organization has developed programs such as the Behavioral Rehabilitation Center for canine victims of cruelty to try and counter fear and anxiety developed in these dogs [77]. It has also been reported that other negative behaviors, such as aggression, fear, repetitive and attention-seeking behaviors, are at an increased incidence in dogs subject to cruelty [78]. This was determined using the Canine Behavioral Questionnaire as an assessment tool.

## 5. Other Welfare Issues Arising from Dog Fighting Practices

The animals used in dog fights are exposed to both physical and psychological harm. The types of damage inflicted can include abuse during breeding, or by performing tail and ear docking to prevent their opponents from hanging on to these zones during fights and to generate a more aggressive body language. Sharpening the teeth with a file, starvation, beatings, torture, and social isolation are also used to generate aggressive behaviors.

The training of fighting dogs begins when they are one year old. They are introduced into this activity gradually, first in “informal” fights where they are selected for their aggressiveness or ability to defend themselves. Later, they are made available for organized dog fights in accordance with their weight and sex (though males are usually preferred) [47]. To increase their size, aggressiveness, resistance, and strength during fights, dogs are exposed to training with weights or on treadmills. This activity often causes abrasions on their paw pads or the axillary regions due to the continuous use of the weight vest [39].

Another implication of dogfighting training on their welfare includes the administration of anabolic steroids (e.g., testosterone propionate) to promote muscle mass growth and strengthening [2,79]. Owners may use illegal narcotics (e.g., ephedrine, cocaine, and methamphetamine) or substances, such as gunpowder and hot sauce, to increase the aggression of animals and reduce the perception of pain during the fight [47,80]. These drugs are used without medical supervision, ignoring the serious cardiovascular consequences this may arise [81].

Dogs that will be trained to fight may be bred, bought, or stolen. From the outset, they lack basic care or are subjected to abuse. For much of their life they may be chained or caged [82], only having contact with humans or other animals when they are let out for training or taken to a fight. Moreover, they are generally fed inadequately, are not allowed free access to water, and may not have shelter [83]. Part of the training to make them aggressive fighters involves forcing them to confront wild animals, cats, rabbits, stray dogs, or dogs that breeders steal to use as prey during training [1]. The use of shock or prong collars, hanging the dogs, and severe corrections with choke chains are usual practices during the training of dogs [84]. Likewise, pulling weights and being starved and burned with lit cigarettes, or beaten with several objects to increase their strength and aggressivity are also elements that may cause injuries, tendon and ligament ruptures, persistent muscular pain [79], and emotional discomfort that affects the welfare of dogs [85]. Indicators of negative mental states in dogs include spinning and circling, repeated bounding and rebounding, excessive licking with the formation of granulomas, excessive chewing of objects, and digging [86,87]

Veterinary treatment performed by amateurs is another welfare cost, since physical lesions, such as puncture wounds, lacerations, blood loss, dehydration, shock, and bone fractures may become infected and deteriorate the overall health of the animal. Finally, dogs that have been retired from fighting are often simply killed by being shot or hanged [3].

It is now recognized that for an animal to experience good welfare a greater number of positive emotions (pleasure, happiness) should be experienced than negative ones, such as fear and pain [88]. An animal’s emotional state plays a key role in its behavior, communication, social bonding, and cognitive functioning [89,90]. The environment in which fighting dogs are raised can lead to negative emotions, such as fear, anxiety, aggression, suffering, rage, frustration, and deception [87,91], generating an overall negative affective state. This may further lead to the development of long-term behavioral issues as a result of the trauma, such as fear of their surroundings, continuous barking at conspecifics, or fear-aggression [27].

Dogs trained for fighting do not suffer only physical pain but emotional responses. Whilst there are no direct data on physiological responses to these events in fighting dogs, based on studies in other species, it is plausible that a state of constant stress is generated that triggers innumerable physiological reactions. Reactions of this kind recognized to date include hematological alterations (erythrocytosis), the increased production of peroxides and lactic acid that reduces the accumulation of muscle glycogen and pH [60], and large quantities of enzymes that pour into the bloodstream, including creatinine kinase (CK), lactate dehydrogenase (LDH), and aspartate aminotransferase [92].

In humans, the endogenous opioid system contributes to the processing of emotions, perhaps by differentially influencing specific basic emotions or by modulating the activity of the lower-order system [93]. Humans, of course, can report experiences of suffering and pain through language. In animals, in contrast, pain can only be inferred through physical reactions [94], behavioral changes, such as maladaptive acts [51], and by observing facial expressions [95]. However, studies of animal behavior, cognitive psychology, and functional brain imaging suggest that painful affectations and pain memory are highly probable in animals [94].

## 6. Profile of the Breeders and Handlers of Fight Dogs

Criminological analysis has suggested there are three types of breeders of fighting dogs: those who raise dogs as street fighters, those classified as hobbyists (i.e., for entertainment or as a pastime), and professionals [96]. Those in the first group use techniques such as depriving the dogs of food, administering drugs, and subjecting them to various kinds of abuse [97]. They use their dogs for spontaneous—not organized—fights that are often held in people’s yards or garages with exchanges of money or drugs. The hobbyists make their dogs fight simply for entertainment purposes. They usually have only one or a few dogs which they make available for organized fights [2]. Professional breeders make enormous amounts of money from dogfights and carefully breed their champions. They usually raise many dogs, perhaps 50 or more [13,98]. When these dogs fail to win, or if they flee from the ring, their breeders often kill them by strangulation, electrocution, gunshots, or hanging [98].

There may be linkages of dog fighting with other types of crimes. It is not uncommon for breeders of fighting dogs to be detained on other charges, often related to drugs, alcohol, illegal weapons, corruption of minors, money laundering, or even rape and murder [1]. Kalof and Taylor [99] reported that some ninth-graders they interviewed in a secondary school in Pontiac, Michigan, admitted that they had attended dogfights. Most of them stated that there was nothing wrong with those events. This led the authors to conclude that those children were desensitized to violence to such a high degree that they did not perceive dogfights as cruel, immoral acts [99]. There is also a risk that exposure of young people to these organized acts of animal abuse may lead to later development of behavioral traits that include animal abuse as well as bullying and delinquency [100].

From the handler’s perspective, they may justify dog fighting since many of the breeds involved have been raised for this purpose. They affirm, moreover, that it is in the nature of this breed to fight and combat and that if opportunities to do so are withheld they can experience frustration [4,101]. According to Hartman et al. [102], abuse towards animals is an antisocial behavior and one of the principal criteria for diagnosing behavioral disorders. Likewise, Burley [103] mentions that behind the minds of young people who keep and attend dogfighting events, there is a feeling of self-support and the thinking that a dog who fights is honorable. In extreme cases, dogfighting is associated with illegal drug trafficking, and the aggressiveness of fighting dogs represents an extension of their social status within a group [99].

## 7. Rehabilitation for Fighting Dogs

Rehabilitating these animals is particularly difficult because they have been trained throughout their lives to attack, bite, and kill other animals. As a result, they tend to be very unbalanced and extremely dangerous for both other animals and humans. For this reason, every year in the United States approximately 4 million dogs used in organized fights end up in shelters and are euthanized because it is impossible to readapt them and place them in homes due to their aggressive tendencies [104]. There are, however, cases of dogs that are not severely unbalanced, where it has been possible to demonstrate that they do not present aggressive behaviors towards other animals or humans, despite having been trained as fighters [15]. This has fostered the development of programs that seek to achieve the reintegration of fighting dogs into adoptive homes once they show that they have learned to co-exist with humans and other animals [105]. These efforts are related to the field of animal welfare, which is being studied in ever greater detail. Animal welfare does not seek only to maintain animals in healthy and well fed states but also to attend to such crucial aspects as their emotional wellbeing and mental health through, for example, studies of positive and negative emotions [106,107]. Further, the use of formal behavioral assessments in animal shelters is now common in an attempt to identify behaviors that may preclude rehoming. Whilst there is some concern about the ability of formal tests to predict future behavioral issues, especially aggression, the case of dogs with a history of fighting is a compelling reason to accelerate progress in this area.

## 8. Legal Aspects

Since 2009, dog fights have been considered a felony in all 50 U.S. states [2], as is trafficking dogs for this purpose [46]. The maximum penalty for this practice being three years in jail or a USD 250,000 fine. The enforcement of the Animal Welfare Act of 1966 is one of the duties of the United States Department of Agriculture (USDA) with the American Society for the Prevention of Cruelty to Animals (ASPCA) playing an advocacy role around legal reform. The ASPCA refers to this practice as a blood sport and a form of animal cruelty and, in the past decade, it has assisted in more than 200 cases of dogfighting with more than 5000 victims [108]. The ASPCA recognizes that dog fighting is also associated with other inhumane techniques, such as ears cropping and tail docking, frequently performed by the owners [109]. In February 2014, the Farm Bill was signed into law—this included the Animal Fighting Spectator Prohibition Act, a statute aiming to deny the entry of minors under 16 years of age to any event related to animal fighting [96]. The Act is notable in not only targeting those who stage fights but spectators who are ultimately creating the incentive for them.

More recently, the Federal Bureau of Investigation (FBI) began to track any act of animal cruelty, including fighting, as a felony alongside arson, burglary, assault, and homicide [110]. Particularly, the National Incident-Based Reporting System (NIBRS) recognizes that animal cruelty can be a precursor to other offenses or interpersonal violence; therefore, the participation of these associations responds to animal and social welfare [110,111]. Although some programs in the U.S. encourage the rehabilitation of these animals, the cost and complexity of the process ends with euthanasia in most cases [112].

In England and Wales, dog fights are not classified as an independent crime, as seen in the US [113], but Section 8 of the 2006 Animal Welfare Act contains the “animal fighting” offence, where dog-fighting, cockfighting, bear baiting, and badger baiting is included, and is designed to consider these animals as protected against suffering. Dog fighting is prohibited in the UK, and animal abuse is punishable by a fine and can lead to up to 12 months to 5 years, depending on the severity of the offence [114].

There are also restrictions on possession, breeding or selling of certain breeds of dog, under the Dangerous Dogs Act of 1991. The breeds Argentine Dogo, Pit Bull Terrier, Brazilian mastiff, and Japanese Tosa Inu are often acquired for fighting [3,97]. Many of these same breeds are also subject to importation bans in other countries, for example Italy [115]. The challenges in enforcement due to the need for reporting of the act, as well as adequate resourcing and evidence collection have been acknowledged.

The RSPCA holds a Special Operations Unit in charge of monitoring and investigating organized animal cruelty, such as animal fighting (including dogfighting), an activity considered illegal for 180 years [116]. The SSPCA is the RSPCA’s Scottish counterpart, dedicated to investigating, gathering evidence, and taking persons of interest to court for dog fighting and related offences [117]. LACS works together with other institutions to raise awareness toward illegal animal fights, directing their efforts towards veterinarians, politicians, and society. One of their approaches is through proposals advocating for law reform to consider dogfighting as a distinct crime [118]. This may serve to increase focus and awareness of this crime, increasing the deterrent value and highlighting legislative intent to the judiciary, potentially increasing the sentencing severity for this act of cruelty.

Australia has approached the issue similarly with all states prohibiting animal fighting within their state and territory animal protection statutes [119]. Additionally, as Nurse [97] reports, although some countries may not recognize dogfighting as an actual offense per se, this activity is frequently associated with other actions that are stipulated as crimes by law and can be prosecuted. For example, illegal gambling and weapons, drug dealing, money laundering, the illegal practice of veterinary medicine, or aspects regarding animal cruelty or a diminished welfare [22,112]. However, a disadvantage of this as an alternate avenue for dealing with this crime is that lack of a specific offence may not provide the required deterrent effect, and these other crimes cross many areas of law with differing enforcement agencies increasing the complexity of enforcement. UK law specifies related acts that are categorized as crimes, including possessing or training dogs for fights, organizing, participating in, or attending dogfights, forming a business, betting on them, and tail docking [93,94,120,121]. There are also aggravated offences depending on the degree of offense or involvement, be it active or passive participation. Active offenses are those in which a person benefits and directly engages in dogfighting; in contrast, passive activities include those where the person does not directly participate but organizes, promotes, or bet in fights [122].

In the Netherlands, dogfighting has been associated with organized crimes that are part of an illegal market and usually related to animal abuse, brutality, and felony [123]. The banning on organizing and participating in animal fights is part of the Animal Health and Welfare Act, Article 61. Particularly, Article 2.14 of the Animal Law Act refers to dogfighting and stipulates the time of imprisonment (no more than three years) and fines [79]. In France, acts of cruelty against animals, including abandonment, carry penalties of two years in prison and a fine of up to EUR 30,000. In Italy, all forms of animal torture are punishable by one year in prison and a fine. Abandonment, keeping animals in small cages, and malnutrition are considered acts of animal torture that are also seen in dog fighting, and in Switzerland, the cruel treatment of animals is punishable by up to three years in prison and a fine of CHF 20,000 [123,124].

As reported by Rossi-Broy [125], in Germany there are federal regulations prohibiting the ownership of breeds of dogs referred to as “dangerous dogs”. These breeds have been previously used for dogfights, and when trying to reinsert these animals into homes, it has become almost impossible, so they end up being taken to shelters. Among the breeds of dogs that are prohibited in almost all states of Germany are American Staffordshire, Bull Terrier, Pitt Bull Terrier, and Staffordshire Bull Terrier. Additionally, in Germany, all dogs must be registered with the City Council and their owners are obliged to pay a tax for keeping the animal [126]. In this country, anyone who kills a vertebrate animal for no reason can be sentenced to three years in prison [123].

In the case of Latin American countries, chapter VII of the Animal Protection Law enacted by Mexico’s Legislative Assembly deals with the dignity of, and respect for, animals. Article 24 of the law prohibits training animals to attack other animals or humans and turning the fights so provoked into public or private spectacles [127]. In 2017, Mexico’s Penal Code approved up to 7.5 years in prison for people who participate in dogfights [128]. Unfortunately, there is little evidence that these sanctions will be applied any time soon. According to Pereira [129], in Chile, only 21.30% of municipal regulations prohibit any activity associated with the training and partaking in dog fights. However, the Animal Protection Law (Law 20,380, 2009) and the country’s Penal Code interpret fights as an act of animal abuse even though it is not marked as such by the law, so its severity and designation depend on the judges’ criteria [129].

In 1900, Law 3959 of the Animal Sanitary Police was approved in Argentina to include animal fights in their regulation (e.g., dog and bullfighting), with a prison sentence of 15 days to one year. In Bolivia, the Law for the Defense of Animals Against Acts of Cruelty and Mistreatment considers that animals have the right to be recognized as living beings, so they must be protected from any type of violence. Colombia also recognized sentient animals in 2016, in Law 1774, similar to what is reported in Guatemala, Nicaragua, and Peru. In Costa Rica, Articles 279 of the Penal Code punish cases of animal abuse and fights between animals with sentences of up to one year in prison. In Ecuador, the sentences do not exceed ten days of the deprivation of liberty, but if the fights cause mutilation, injury, or death of the animal, it will be sanctioned with a custodial sentence of fifteen to thirty days [124].

In contrast, in some Asian countries, such as Afghanistan, dogfighting is one of the most popular sports in the country [130]. Although it is banned by the Taliban, people see it as recreational activity and the fights are policed by authorities to ensure the safety of viewers [131]. Similarly, in southern Punjab, Pakistan, far from having laws that prohibit them, dogfighting is considered as a cultural activity, and people participate in it enthusiastically, especially within the Punjabi culture, where it is perceived as a synonym of masculinity and honor of the fighters [12]. In another Asian country, Japan, dogfighting was considered legal in ancient times as a national tradition and tourist attraction associated with samurai and similar to sumo wrestling, using Tosa breed dogs, which are highly prized [79]. To date, this practice has not been banned throughout the territory, and only five prefectures (including Tokyo and Hokkaidou) have laws forbidding dogfighting [131]. Similarly, in China, dogfighting remains legal as a historical tradition of the country [132].

Regardless of the country, most have laws that penalize breeding, buying, selling, keeping, transporting, organizing, promoting, and attending dog fights [133]. The breadth of legal responses to this issue shows that there are clear cultural and historical aspects to this topic that may not be first apparent. Nonetheless, it is important to recognize that punishments and the inclusion of dog fighting into a legal framework follow ethical concerns that depend on the concept of sentience. Law enforcement and advocacy can be carried out by institutions that promote animal welfare, such as the ASPCA or the Humane Society of the United States, which organize actions against dogfighting [132]. These associations help to prioritize dogs’ welfare by raising awareness in society.

## 9. The Potential Role of the Veterinary Profession in Dog Fighting

Dogs are frequently taken for veterinary attention by handlers who give false accounts of how the injuries occurred [134], saying, for example, that they happened during a spontaneous or unexpected fight with another animal. This is one reason why veterinarians often do not report situations to the authorities. A second reason is that few veterinarians have been trained to detect these signs [5,8]. A third more fundamental, and often contentious, issue is the need for mandated reporting of animal abuse by veterinarians. Many countries do not have such a requirement and indeed it may conflict with professional guidelines around confidentiality. The main concern around implementing this being that owners may not seek veterinary guidance and welfare may be further compromised [135]. However, there are also likely professional barriers, such as fear of litigation from reporting. In light of this, there have been calls for statutory protection of vets who report animal abuse.

It is difficult for veterinarians to intervene if they are not sure that the lesions in question were caused by an organized dogfight. Their role is complicated by the fact that the victims cannot speak for themselves, and that all information given during veterinary consultations is protected by doctor–patient (in this case, owner) privilege [136]. Nor can we ignore the reality that veterinarians may fear retaliation by individuals dedicated to organizing dogfights because of their possible criminal connections. In other cases, they simply may not know which authorities could provide support. Veterinarians are, however, obliged to act in favor of the wellbeing of animals at all times, and there is likely an ethical obligation to report abuse if a suspicion arises [3,137]. This in the recognition of preventing further welfare detriment and the well-recognized link between human and animal violence. It is important that veterinarians collaborate in forensic medicine with the police, other enforcement agencies, and pathologists to determine the cause of the lesions they identify and, in this way, detect illicit acts [138]. They also need to ensure that that their records and evidence collection meets a high standard to stand up to court scrutiny and be prepared to be called upon as a witness in court.

For all these reasons, veterinarians rarely—or never—report cases. Thus, it is essential to develop undergraduate courses, seminars, or workshops to train veterinarians in the detection and adequate handling procedures for cases of animal abuse [106]. Additionally, veterinarians could educate society and dog owners on dog fighting—encouraging reporting of this activity to the relevant authorities since it often occurs in the suburban backyard. These actions, together with more severe sanctions and control, established by the law and authorities, could help to share the responsibility towards the problem of dog fights.

## 10. Perspectives and Final Thoughts

Although interest in animal welfare is growing, there is still widespread abuse of companion animals and dog fighting is one of the more extreme examples. Since the resourcing of enforcement authorities is often an issue and abuse may be relatively hidden, it is important to educate the public and veterinarians on this issue and signs of it occurring develop more effective programs for the timely detection of animal mistreatment to contribute to preventing this behavior and to opportunely denouncing people who are dedicated to organizing dogfights. The law arguably should also act in a deterrent role by denouncing such acts, and this may require strong messages in the form of maximum penalties in the written law and being handed down in courts.

There is also further research needed on how to best reintegrate former fighting dogs into a home environment and, in case this is possible, to improve the programs of rehabilitation. Other educational activities for adults and children with input from veterinarians would also be of benefit. For children, education in schools should include lessons about the basics of responsible ownership of companion animals and impart the importance of empathy towards all living beings [139]. Furthermore, education for veterinarians should not be limited to a clinical perspective. It also needs to focus on understanding the importance of maintaining and promoting animal welfare in any practice where animals are under human care [106]. This includes the concept of responsible pet ownership, which not only promotes strategies for the care and prevention of pet abandonment but also awakens society’s conscience about companion animals’ well-being [140]. The science of animal welfare includes satisfying the biological needs of animals but also understanding that, through non-verbal communication, such as facial expressions or body language, dogs are capable of transmitting emotions [141]. The goal would be to generate a mentality of respect and love for the planet and all the beings that inhabit it.

## 11. Conclusions

The content of this review establishes that the environment that surrounds dogs raised for fighting is unacceptable, because the animals are continuously abused; fighting dogs suffer actions that jeopardize their physical and emotional health. For their impact on dog welfare, such activities must be discouraged by a legal ban. It is clear, however, that there are cultural and historical factors that may make this unlikely in some countries/regions. Proactive efforts must be made to educate citizens on the welfare impacts of dog fighting and the requirements of reporting it. A second area of vital importance is to train veterinarians to detect, identify, and accurately diagnose injuries inflicted during organized dog fights, and to distinguish between spontaneous and organized fights. This may require guideline development that specifies how, when, and where they can act to prevent or report cases of animal abuse. Much remains to be done, as well, to ensure the enforcement of existing laws as a way to improve animal welfare and to provide veterinarians with the tools they require to act efficaciously in these cases.

## Data Availability

Not applicable.
